# Anteromedial Surface Plating for Midshaft Fracture Humerus Through an Anterolateral Approach - A Better Option than Anterolateral Plating

**DOI:** 10.5704/MOJ.2011.011

**Published:** 2020-11

**Authors:** SK Rai, AD Sud, M Kashid, B Gogoi

**Affiliations:** 1 Department of Orthopaedics, Indian Naval Hospital Ship Asvini, Mumbai, India; 2 Department of Orthopaedics, Armed Forces Medical College, Pune, India; 3 Department of Orthopaedics, SMBT Institute of Medical Sciences and Research Centre, Nashik, India; 4 Department of Orthopaedics, 151 Base Hospital, Guwahati, India

**Keywords:** medial surface plating, humerus shaft fractures, anterolateral approach, radial nerve palsy

## Abstract

**Introduction:**

Osteosynthesis by plate fixation of humeral shaft fractures as a gold standard for fracture fixation has been proven beyond doubt. However, during conventional anterolateral plating Radial nerve injury may occur which can be avoided by applying plate on the medial flat surface. The aim of this study was to evaluate the results of application of plate on the flat medial surface of humerus rather than the conventional anterolateral surface.

**Materials and Methods:**

This study was conducted between Oct 2010 to Dec 2015. One-hundred-fifty fracture shafts of the humerus were treated with the anteromedial plating through the anterolateral approach.

**Results:**

One-hundred-fifty patients with a fracture shaft of the humerus were treated with anteromedial plating. Twenty were female (mean ±SD,28 years±4.5) and 130 were male (mean ± SD, 38 years±5.6). One hundred and forty-eight out of 150 (98.6%) patients achieved union at 12 months. Two of three patients developed a superficial infection, both of which were treated successfully by antibiotics and one developed a deep infection, which was treated by wound debridement, prolonged antibiotics with the removal of the plate and subsequently by delayed plating and bone grafting.

**Conclusion:**

In the present study, we applied plate on the anteromedial flat surface of humerus using the anterolateral approach. It is an easier and quicker fixation as compared to anterolateral plating because later involved much more dissection than a medial application of the plate and this application of plate on a medial flat surface, does not required Radial nerve exposure and palsy post-operatively. The significant improvement in elbow flexion without brachialis dissection is also a potential benefit of this approach. Based on our results, we recommend the application of an anteromedial plate for treatment of midshaft fractures humerus.

## Introduction

Osteosynthesis by plate fixation is the accepted and well-documented surgical procedure of internal fixation for diaphyseal diaphyseal fracture of the humerus using anterolateral approach and is widely used. However, the outcome of humeral shaft fracture fixation using a medial approach is not much discussed in the present literature. The aim of this study is to explore the management and outcomes of the application of medial plate in humeral mid-shaft fractures through a standard anterolateral approach.

Fracture shaft of humerus account for 1.2% of the majority of cases seen in the orthopaedics as well as in the emergency department1. As we all know that humerus shaft extends from pectoralis major insertion to supracondylar ridge. Attachments of pectoralis major, deltoid, and rotator cuff muscles influence the fracture segment displacement. Simple and undisplaced fractures of shaft of humerus can be treated conservatively by plaster immobilisation. However, it has own disadvantage like angulation and displacement later on. Plate fixation or osteosynthesis remains the gold standard of surgical treatment. Although the plate fixation results in high rates of fracture union but require extensive dissection and soft tissue stripping. Denies *et al*^2^, in his study of 91 patients showed medial plate fixation was better than intramedullary nailing. Livani *et al*^3^ in their study showed anterior plating is a simple, safe, and effective treatment for humeral shaft nonunion. Antero-Medial placement of the plate avoids the requirement of unnecessary radial nerve exploration, retraction, and thus extensive soft-tissue dissection.

The most commonly used approaches for treating distal 1/3rd shaft fractures are posterior and sometimes anterior. Boschi *et al*, described Subbrachial approach to Humeral shaft fractures4.

A per literature, the overall incidence of the radial nerve injury through posterior approach is 11% and by anterolateral approach is 5.4%.

## Materials and Methods

This is a prospective study of 150 patients where medial surface plating was done for the mid shaft fracture of humerus between Oct 2010 to Dec 2015. Before this study, these fractures of midshaft humerus were treated by conventional anterolateral plating and their data were used as control group. A total of 85-fracture humerus were plated in conventional anterolateral way named as Anterolateral plate group in present study. Fracture exposure time, blood loss, requirement of radial nerve exposure, post-operative radial nerve palsy and requirement of plate contouring was compared with previous recorded data. One-hundred-fifty patients consist of young soldiers and their family members (120 male and 30 female patients) (Table I) with a midshaft humerus fractures were included and treated with antero-medial plating through standard anterolateral approach.

**Table I:** The characteristics of patients

Characteristics	No of patients
Number of cases	150
Gender (male/female)	130/20
Mean age, years (range)	19-65
Mechanism of injury	
Road Traffic accident	25
Fall during military training and duty	76
Sport injury	36
Injury during fall at home	13
Fracture type (AO/OTA Classification)	
Type A	101
Type B	21
Type C	28

The patients of non-union of fracture Humerus or any previous surgery on the same part, patients with pathological humerus fracture, infected non-union, gap non-union and patients presented with radial nerve injury/palsy were also excluded from the study. Included patients were followed for a period of minimum six months and maximum up to two years. Injury during duties and training accounted for 50% of the cases. All patients were operated within three to seven days following injury. All patients were given general anaesthesia and were placed in supine position. Our skin incision begins from the lateral edge of the biceps brachii muscle and follows its border, avoiding the cephalic vein till 5cm above flexion crease of elbow.

We used this approach, in which the whole brachialis muscle was spared and act as protection between the operative field and the radial nerve. The separation of the biceps and the brachialis muscle start proximally. With this sub-brachialis approach there is absolutely no requirement to expose the radial and the musculocutaneous nerve because they are outside the operative field. Subsequently, the biceps brachii and the brachialis muscles were exposed by retraction of skin and fascia.

In the anterolateral approach, the brachialis muscle split into two sleeves, which was then, retracted laterally and medially however in sub-brachial approach used in this study, the whole muscle was retracted laterally, following the edge of the brachialis muscle and isolating it from the biceps brachii muscle’s lateral edge toward the humerus using a blunt finger dissection. The plane between these two muscles starts proximally and in order to avoid the musculocutaneous nerve damage, dissection done using a finger or periosteal elevator. The humerus can be exposed from the place where the medial part of the brachialis muscle is loosely attached to the bone. From there, the muscle isolation procedure can be performed distally (for the middle and the distal third of the humeral diaphysis). The muscle was isolated by a blunt finger dissection. At this point of time, the whole brachialis muscle can easily be retracted and moved laterally. By doing such dissection described supra, we exposed anteromedial surface of the humerus which is more smooth and nearly flat to accommodate plate.

**Fig. 1:**
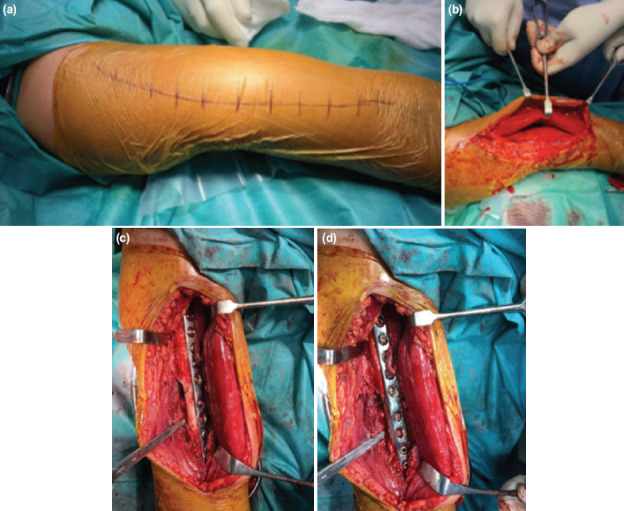
Medial plating is being done and application of plate on flat medial surface of humerus. Clockwise – (a) skin incision, (b) biceps retracted laterally, (c,d) brachialis was elevated subperiosteally and plate applied.

**Fig. 2:**
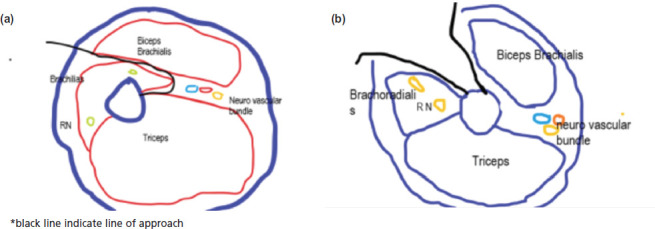
(a,b) Diagrammatic presentation of sub brachialis approach for medial humerus plating.

Finally, the Brachialis muscle was made free, elevated from the humerus and retracted laterally to visualise the fracture ends similar to standard anterolateral approach (Fig. 1). The flat medial border of humerus was then exposed, using periosteal elevator. Since all dissection done on medial aspect of humerus, exposure of radial nerve was not required. Fig. 2 shows diagrammatic presentation of sub-brachialis approach for medial humerus plating. After reduction of the fracture, 8/9 hole 4.5mm narrow titanium locking compression plate was applied using standard AO technique (in 30 patient) and dynamic compression plates 8/9 hole 4.5mm narrow in 120 patients, and wounds were closed in layers. No suction drain was placed.

**Fig. 3:**
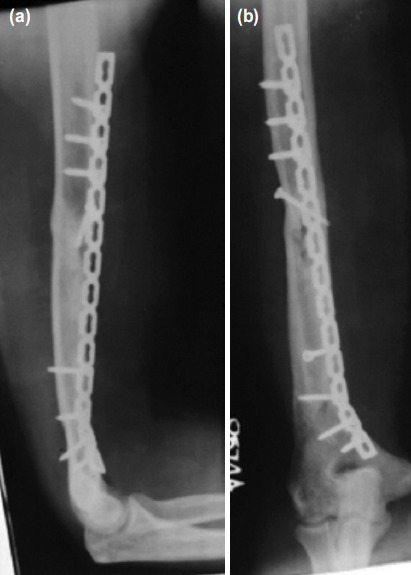
(a,b) Comminuted segmental fractures midshaft humerus (AO type C2 segmental) and application of medial plate. 12 months post-surgery. (Please note: In case of distal extension of midshaft fracture, a plate needs to be contoured before application.

Once patient became pain free, passive and active shoulder range of motion and elbow exercises were started from second post-operative day. Radiographs were assessed at 12 weeks, 18 weeks, 6 months, 9 months and 12 months for fracture union. However, it is not advisable to apply medial plate exclusively for distal 1/3rd shaft fracture unless it is an extension of midshaft fracture (Fig.3).

## Results

One-hundred-fifty patients with fracture shaft of humerus were treated with the anteromedial plating. Twenty were female and 130 were male. Two out of three patients developed a superficial infection, both of which were treated successfully by antibiotics and one developed a deep infection, which was treated by wound debridement and prolong antibiotics with plate removal and delayed plate fixation with bone grafting. One hundred forty-eight out of 150 (98.6%) patients achieved union at 12 months. One patient showed feature of delayed union at the end of 12 months, which was treated by bone grafting, and the union was achieved at the end of 18 months. Another patient who developed a deep infection and showed non-union even at the end of 16 months was managed by wound debridement and removal of implant. This patient was further treated by plating and bone grafting after 6 months of control of infection and union was achieved at the end of 24 months. We did not note any Radial nerve palsy in any of the patients after surgery. Soft callus (healing) started appearing between 6 to 18 weeks. We did not encounter any metal implant failure in our study. A comparison including fracture exposure time, amount of bleeding, post-operative radial nerve palsy is shown in Table II. Fracture exposure time and amount of blood loss is significantly less in application of plate on flat medial surface as compared to application on anterolateral surface (p-value 0.05). The continuous variables were analysed between anterolateral plate groups and medial plate group using Independent sample t test. P value of <0.05 was considered statistically significant.

**Table II:** Comparison between application of plate on flat anteromedial surface vs application of plate on anterolateral surface

Humerus Surface	Number of patients (n)	Fracture exposure time (mean)	Blood loss in dissection (Mean)	Radial nerve exposure required (n)	Features of Post- operative Radial nerve palsy (n)	Plate contouring required (n)	P-value
Anteromedial – Flat surface	150	24 min	50 ml	nil	nil	nil	0.05
Anterolateral – slight convex surface (Control group)	85	47 min	110 ml	23	03	86	0.05

**Fig. 4:**
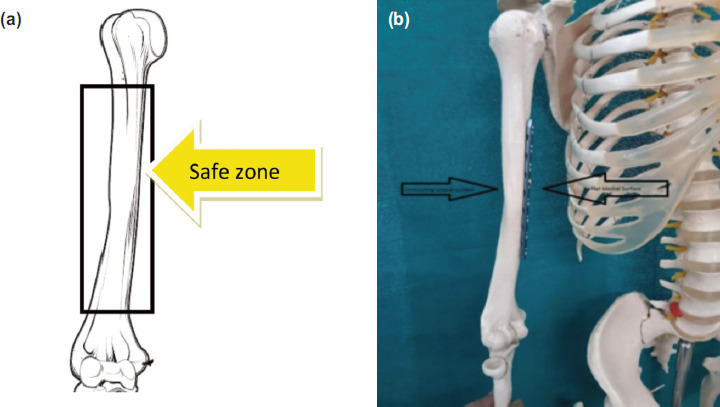
(a,b) Diagrammatic presentation of the safe zone of humerus, where plate can be apply medially through the anterolateral approach. Lateral surface is undulation whereas Medial one is flat.

We measured shoulder and elbow range of motion and muscle power after surgery in all 150 patients after four weeks of surgery when they become totally pain free. One hundred forty-seven patients had full muscle power 5/5 and full range of motion in shoulder and elbow. Two patients had 15° of FFD in elbow and one had 20° because both patients had developed post-operative infection (Table III). We could not compare muscle strength between two groups (anterolateral and medial plate), as no such data is available with us. There was no radial nerve palsy, musculocutaneous nerve complication or brachial plexus injury in the present study. Three patient developed infection, which was managed with appropriate antibiotic therapy. There is a medial safe zone of humerus, where plate can be applied medially through the anterolateral approach. Lateral surface is uneven whereas medial one is flat. Hence, anteromedial plating can be done through an anterolateral incision safely in the midshaft zone of humerus without any complication (Fig. 4).

**Table III:** Shoulder and Elbow range of motion and muscle power after surgery

No of patients	Shoulder power (MRC grade)	Shoulder- ROM	Elbow power - flexion	Elbow ROM	Reason
147	5/5	full	5/5	full	No infection
02	4/5	full	4/5	15° FFD	Developed superficial infection post-operatively
01	5/5	full	3/5	20° FFD	Developed deep infection post-operatively

## Discussion

Due to development in surgical techniques and better implants, fracture shaft of humerus is being treated by surgical means with good results. A study by Denies *et al*^2^ compared conventional plating with intramedullary interlocking nail in 91 patients and showed that there were significant number of complications present in nailing group as compared to plating group.

The posterior and anterolateral approaches are still most common and widely used. Kirin *et al*^5^ in their study recommended anteromedial plating of humeral shaft fractures through anterolateral approach as simple, safe, effective and also fast surgical treatment. In using medial approach, the plate can be place on flat medial surface of humerus without the need of contouring. As per AO guidelines, the plate should be place on the tensile surface. The plate should therefore be place on either the anterolateral or the posterior aspects in case for humerus fracture^6,7^.

Another study by Oh *et al*^8^ and Luthar *et al*^9^ where they compared open reduction with minimally invasive plate osteosynthesis in humeral shaft fractures and both reported no significant difference in fracture union. Boschi *et al*^4^ in his study concluded that the sub-brachial approach as practical and effective and the loss of muscle strength was significantly less with sub-brachial approach.

In our study, the medial plating done through anterolateral approach without splitting the Brachialis, the Brachialis muscle was elevated and retracted laterally and there is no need to exposed Radial nerve.

The advantages of medial surface plating over posterior surface plating is supine position of the patient, administration of anaesthesia is easier, no need to expose or handling of radial nerve as it is out of surgical field and less soft tissue dissection and bleeding. Incidence of radial nerve palsy in posterior plating has been reported to be 11.14%10. In our study, we did not notice any radial nerve palsy postoperatively. Kirin *et al*^5^ in his study compared anterolateral plating and anteromedial plating and reported 5.4% of radial nerve palsy when the plate was applied on the lateral surface than on anteromedial surface.

In the literature, whether the radial nerve should be expose or not is a topic of discussion. Many studies and observations have reported that majority (75 - 80%) of radial nerve injuries recover spontaneously as they are neuropraxia due to retraction or nerve contusion following initial injury, and early detection is recommended to reduce iatrogenic radial nerve injuries and litigation later on. To apply plate on anterolateral surface, it required plate contouring and frequent elevation of the deltoid insertion from lateral surface of humerus. In our series, plate contouring was not required as it was applied to relatively flat medial surface, which enables plate to sit on flat surface and thus decreases the operating time. In a study on humerus shaft non-union treated with vascularised fibular bone graft, Jupiter suggested medial approach and application of plate anteriorly.^11^ More recently Kumar *et al* reported on 54 patients that the medial surface plating gives good result without any radial nerve palsy and has reported that it takes much less time than conventional anterolateral plating^12^.

Plate removal is easier than anterolateral. In few cases, patients require plate removal because of indication like hypertrophic non-union, implant failure or because of infection. In these cases, where anterolateral plate has been applied where radial nerve is in direct contact with the plate, local soft tissue scaring and in the presence of bony callus makes plate removal difficult and increased rates of iatrogenic radial nerve injury. However, when plate was applied to the medial surface there is no danger of the radial nerve injury.

There are many advantages of anteromedial plating over conventional anterolateral plating, (a) easier to apply plate on flat medial surface than uneven anterolateral surface of humerus (b) surgical dissection is safe, (c) almost no chance of iatrogenic radial nerve injury, (d) less surgical time than conventional anterolateral plating, (e) less bleeding as no muscle splitting is involved as compared to conventional anterolateral plating, (f) no plate contouring is required as medial surface is flat as it is required in conventional anterolateral plating, (g) comparable outcome with conventional anterolateral plating and (h) removal of plate is easier and there is no chance of radial nerve injury.

In our study, we did not find any radial nerve palsy and operating time is significantly less as compared to standard anterolateral plating as later involved muscle striping and more dissection.

The limitation of this approach is far proximal and far distal extension of fracture. If fracture extends far proximal and distal then, plate contouring is required for proper plate application.

For recommendation, furthermore study with larger study population will be required to bring out further strong evidence that anteromedial humerus plating is better option and the same can be applied using anterolateral approach with better surgical and functional outcome. It also minimises the risk of redial nerve injury in middle third - fracture of the humeral shaft.

## Conclusion

In our series, we performed anteromedial surface plating by using an anterolateral approach, there was no Radial nerve palsy, and more so medial surface of humerus is more flattened than lateral surface hence application of plate in more convenient on medial surface. It is easier and quick fixation as compared to anterolateral plating because later involved much more dissection than medial application of plate. The purpose of this surgical approach is to reduce the dissection through brachialis and minimise neurological complication. Based on our results, which includes less operating time, less tissue dissection, easy application of plate, we can now recommend anteromedial surface plating for treatment of humeral mid shaft fractures.
